# Genes Coding Industrially Relevant Enzymes in Fungi: Isolation and Protein Engineering of Laccases

**DOI:** 10.2174/138920211795564395

**Published:** 2011-04

**Authors:** Vincenza Faraco

It is my great pleasure as a Guest Editor of the journal Current Genomics to present you with a ‘hot topic issue’ on fungal laccase genes. 

Fungi are sources of several enzymes susceptible of applications in many and diverse industrial segments such as food, beverage, textile, leather, paper and pulp, animal feed and fuel industry. These microorganisms are safe, display efficient growth under industrial production conditions and are able to secrete ample quantities of enzymes. The main fungal enzymes having industrial relevance include cellulases, xilanases, amylases, proteases, lipases, laccases, etc. Among these, laccases are blue multicopper oxidases (MCO), using the distinctive redox ability of copper ions to catalyze the oxidation of a wide range of aromatic substrates concomitantly with the reduction of molecular oxygen to water. Catalytic properties of laccases and their low substrate specificity allow their wide application in several industrial sectors such as pulp and paper, textile and cosmetic industries, for detoxification and decoloration of sewage, in organic synthesis, for degradation of xenobiotics and bioremediation, in production of wood-fiber plates, wood blocks, and cardboard without using toxic linkers, for production of detergents and in elaboration of biosensors.

Much attention has been focused on isolation and recombinant expression of genes coding fungal laccases. Great interest is currently drawn by to development of tailor-made enzyme variants more appropriate for specific applications through protein engineering techniques. 

This issue is aimed at reporting reviews on the main results in isolation of fungal laccase genes and their recombinant expression and on the most significant advances in their protein engineering. The scope of this issue is to provide a compendium on currently available molecular tools for application of laccases from different sources.

Kües and Rühl reported an overview of laccase and other MCO genes encoded in genomes of basidiomycetes, along with a description of their phylogenetic analysis and related functions. Pöggeler described MCO coding capacity of ascomycetes.

Piscitelli *et al.* revised most of the published results on fungal laccase induction, as well as analyses of both the sequences and putative functions of laccase gene promoters. Elucidation of the components and the mechanisms involved in regulation of laccase gene expression is crucial for increasing the productivity of native laccases in fungi.

Maté *et al.* described fungal laccases engineering by directed evolution to improve their functional expression or stability, using *Saccharomyces cerevisiae* as a heterologous host laccase. The low redox potential laccase from *Myceliophthora thermophila* was the first successful example and since then, specific approaches for the laboratory evolution of HRPLs that combine *in vivo* and *in vitro* tools with rational approaches were designed. The evolved fungal laccases mentioned in this review constitute platforms for further protein engineering through directed evolution, principally aimed at generating enzymes that can be used in attractive biotechnological applications. 

Robert *et al.* reported an overview on strategies and results of design of new biocatalysts based on laccases from *Trametes* sp. C30 by protein engineering.

I would like to sincerely thank all the reviewers for their valuable suggestions to improve the quality of review articles. Special thanks to Editor-in-Chief Dr. Christian Neri for encouragement for this special issue. It was a great opportunity for me to interact with scientists from different European countries. I am convinced this issue will be useful for the scientists, academicians, industry professionals and students. I will be looking forward to editing another issue on other industrially relevant classes of fungal enzymes in future.

